# Targeting Antibody Responses to the Membrane Proximal External Region of the Envelope Glycoprotein of Human Immunodeficiency Virus

**DOI:** 10.1371/journal.pone.0038068

**Published:** 2012-05-30

**Authors:** Donatien Kamdem Toukam, Matthias Tenbusch, Alexander Stang, Vladimir Temchura, Michael Storcksdieck genannt Bonsmann, Bastian Grewe, Stefanie Koch, Andreas Meyerhans, Godwin Nchinda, Lazare Kaptue, Klaus Überla

**Affiliations:** 1 Department of Molecular and Medical Virology, Ruhr-University Bochum, Bochum, Germany; 2 Fraunhofer Institute for Biomedical Engineering, Sulzbach, Germany; 3 ICREA Infection Biology Laboratory, Department of Experimental and Health Sciences, University Pompeu Fabra, Barcelona, Spain; 4 Laboratory of Immunology, The Chantal Biya International Reference Centre for Research on HIV/AIDS Prevention and Management (CIRCB), Yaounde, Cameroon; 5 Institut Supérieur des Sciences de la Santé, Université des Montagnes, Banganté, Cameroon; Pasteur Institute of Shanghai, Chinese Academy of Science, China

## Abstract

Although human immunodeficiency type 1 (HIV-1) infection induces strong antibody responses to the viral envelope glycoprotein (Env) only a few of these antibodies possess the capacity to neutralize a broad range of strains. The induction of such antibodies represents an important goal in the development of a preventive vaccine against the infection. Among the broadly neutralizing monoclonal antibodies discovered so far, three (2F5, Z13 and 4E10) target the short and hidden membrane proximal external region (MPER) of the gp41 transmembrane protein. Antibody responses to MPER are rarely observed in HIV-infected individuals or after immunization with Env immunogens. To initiate antibody responses to MPER in its membrane-embedded native conformation, we generated expression plasmids encoding the membrane-anchored ectodomain of gp41 with N-terminal deletions of various sizes. Following transfection of these plasmids, the MPER domains are displayed on the cell surface and incorporated into HIV virus like particles (VLP). Transfected cells displaying MPER mutants bound as efficiently to both 2F5 and 4E10 as cells transfected with a plasmid encoding full-length Env. Mice immunized with VLPs containing the MPER mutants produced MPER-specific antibodies, the levels of which could be increased by the trimerization of the displayed proteins as well as by a DNA prime-VLP boost immunization strategy. Although 2F5 competed for binding to MPER with antibodies in sera of some of the immunized mice, neutralizing activity could not be detected. Whether this is due to inefficient binding of the induced antibodies to MPER in the context of wild type Env or whether the overall MPER-specific antibody response induced by the MPER display mutants is too low to reveal neutralizing activity, remains to be determined.

## Introduction

The HIV envelope glycoprotein complex mediates virus entry into the cell [1] and represents the only exposed viral protein on the surface of the virion. The precursor envelope glycoprotein gp160 is cleaved into two subunits, the gp120 surface protein that interacts with receptor (CD4) and co-receptor (CCR5 or CXCR4) and the gp41 transmembrane protein, which anchors the gp160 into the membrane of the virion. After binding of trimers of the mature envelope proteins to the receptors, gp41 inserts its fusion peptide into the cytoplasmic membrane of the target cell, then collapses to form the stable six-helix bundle, bringing closely together viral and cell membranes [2], [3]. The tryptophan-rich membrane proximal external region then mediates fusion of the two membranes allowing the viral core to enter the cell [2], [4].

The MPER is a hydrophobic and tryptophan-rich portion of the gp41 ectodomain close to the lipid membrane. The structure of MPER peptide in a lipid environment (dodecylphosphocholine micelles), revealed an “L” shaped structure with two alpha helices separated by a hinge region [5]. Most of the hydrophobic residues on the same side of the peptide are imbedded into the phospholipid membrane. In the six helix bundle situation and in the presence of the fusion-peptide proximal region, Buzon and co-workers described a helical rod-like structure on the N-terminal side of MPER with a ∼90° turn of the MPER chain at Asn 687 (numbering according to Gen bank entry AF128126.3). The adjacent Trp-Leu-Trp-Tyr sequence was perpendicular to the rod-like structure [4]. Liu and co-workers described a labile α-helical trimeric structure of the MPER spanning residues 672–693 [6]. These observations suggest that the MPER within the functional envelope spike adopts a complex structure including trimerized and non-trimerized parts as suggested by Zhu and co-workers [7], [8], that may influence the epitope conformation recognized by MPER-targeted neutralizing antibodies.

The MPER is a well-conserved sequence [5] that bears epitopes for three broadly neutralizing antibodies (bnAb) [9], disseminated on a 22 amino acid long peptide. The 2F5 core epitope (ELDKWA) is N-terminal to the 4E10 core epitope (NWFDIT), and the Z13 epitope overlaps both 2F5 and 4E10 epitopes [10]–[13]. The MPER is intensively studied as it represents one of the most interesting targets for HIV vaccine research [14]. Both 4E10 and 2F5 monoclonal neutralizing Abs were obtained from PBMC of HIV-1 infected individuals, while Z13 was obtained from an antibody phage display library prepared from bone marrow of an HIV-1 infected donor [13]. However, antibodies to the epitopes recognized by these three broadly neutralizing monoclonal antibodies are rarely detected in HIV infected subjects [15], [16], a situation that may be explained by several factors including sterical occlusion due to bulky gp120 domains, intensive glycosylation, or immune diversion by more immunogenic decoy structures [17]. When the MPER peptide is expressed as a fusion protein or displayed on various surfaces, MPER peptide-specific antibodies can be induced [18]–[21]. However, results from these studies indicate that these MPER peptide-specific antibodies have rarely neutralizing activity, suggesting that the right conformation of MPER is important [18]. Rather than grafting MPER into heterologous scaffold proteins, we explored in the present study whether one could increase the accessibility of MPER by deleting large parts of the N-terminal regions of gp41. The C-terminal membrane anchorage was preserved in order not to disturb the embedding of the peptide in the lipid membrane.

## Materials and Methods

### Animals

Six to eight week-old female Balb/c mice were purchased from Janvier (Le Genest-ST-Isle, France) and housed in singly ventilated cages in accordance with the national law and institutional guidelines.

### Cloning of the Mutants

MPER mutants were generated by standard PCR amplification techniques, using six different forwards primers that bind the gp41 upstream near the MPER, and a reverse primer that binds the 3′-end of gp41. The clade B-derived HIV-1 envelope R2 [22] in which the intracellular domain was substituted by that of VSV-G was used as PCR template. The open reading frame of the MPER display mutants also encoded at the N-terminus the mouse IL-7 leader peptide followed by a GCN4 isoleucine zipper [23], an Ollas-tag [24] and a G4S flexible linker. Constructs lacking the GCN4 isoleucine zipper were also generated. The open reading frames were cloned between the CMV promoter and the BGH polyadenylation signal of the pVax 200-DEST vector (Invitrogen).

### Transient Transfection of HEK293T Cells

HEK293T cells transfection was performed using polyethylenimine [25]. Briefly, cells were seeded to reach 90% confluency at the time of transfection. For a 75 cm^2^ flask, 2.1 ml of DMEM medium was placed in a 10 ml tube, DNA plasmid was distributed into the medium and mixed. Polyethylenimine (PEI) working solution (1 mg/ml in H_2_O, pH7) was added to a ratio of 1 µg plasmid DNA for 1.5 µl PEI (w/v), the mixture was vortexed thoroughly and incubated 10 minutes at room temperature (RT). The medium in the tissue culture dishes was replaced by fresh medium containing 2% FBS and 0.25 mg/ml Gentamycin, then the transfection mixture was added and the dish was gently swirled, and incubated at 37°C, 5% CO_2_ in humidified atmosphere. Six to eight hours later, the medium was replaced again with fresh medium containing 2% FBS and 0.25 mg/ml gentamycin and cells were incubated for 24 to 48 hours.

### FACS Analysis of Transfected Cells

HEK293T cells were transfected as describe above with the different MPER display mutants or *env* expression plasmids. At 36 hours after transfection, cells were detached from the tissue culture flask using a scraper and gently re-suspended by pipetting up and down. The cells were thereafter pelleted at 800 g for 10 minutes and washed three times with 0.5% PBS-BSA buffer. Subsequently, 2.0×10^5^ cells were added per well of 96 round-bottom-well plate, centrifuged at 2500 RPM and the supernatant discarded. 100 µl of the first Ab (2F5, 4E10 or 3D6, Polymun Scientific, Vienna) diluted in PBS-BSA were added, the cells were resuspended and incubated for 45 minutes at room temperature. The cells were then washed three times, and 100 µl of the fluorescently labeled secondary Ab diluted in PBS-BSA were added, mixed and incubated 30 minutes at RT. Cells were then washed four times and resuspended in 400 µl of PBS-BSA and analyzed using FACS Calibur (Becton Dickinson). FACS data were later analyzed using FlowJo software.

### Production of Virus Like Particles

For virus like particles (VLP) production, transfections were carried out in 175 cm^2^ flasks using 20 µg of plasmids expressing MPER display mutant, gp41 or the R2 *env* and 20 µg of the *gag-pol* plasmid HgpSyn [26]. Supernatants were harvested 36 to 48 hours post transfection, cleared by low speed centrifugation (800 g, 10 minutes at 4°C), filtered through 0.45 µm filter and carefully loaded on the top of 5 ml of 20% sucrose cushion into 25×89 mm open-top Polyclear centrifuge tubes (SETON). The filled tubes were balanced and centrifuged for 3 hours at 25,000 rpm and 4°C in a SW28 rotor (Beckman). Pellets obtained were re-suspended in 250 µl of 1×PBS and stored at −80°C.

### Western Blot Analysis

VLP samples were prepared by adding an equal volume of 2× sample buffer (130 mM Tris-Cl, pH8.0; 20% (v/v) Glycerol; 9.2% (w/v) SDS; 0.02% bromophenol blue; 2% DTT), prior to heating at 95°C for 15 minutes, vortexing for 5 seconds and cooling on ice for 5 minutes. Separation was performed in 15% acrylamide, 0.1% SDS gels for 9 to 12 hours at 50volts and 4°C. For the non-denaturing condition, the SDS was omitted from the sample buffer and samples were loaded without prior heating on 15% acrylamide gel containing 0.1% SDS. After blotting, membranes were blocked for 1 hour at RT in 5% milk (5 g of milk powder in 100 ml PBS-T) under gentle shaking, briefly washed with PBS-T (0.1% Tween 20 in 1×PBS) and incubated for two hours with the primary antibody. Afterwards the membrane was washed three times (3×5 minutes under gentle shaking), incubated 1 hour with goat polyclonal anti-human IgG-HRP (Sigma-Aldrich, Taufkirchen, Germany) and then washed three times. Protein bands were visualized in a luminometer equipped with a Hamamatsu CCD camera (model C2400-75H-01, Hamamatsu Photonics K.K., Japan) after incubating the membrane with reconstituted ECL chemiluminescent solution (Alpha Innotech, San Leandro, USA).

**Figure 1:**
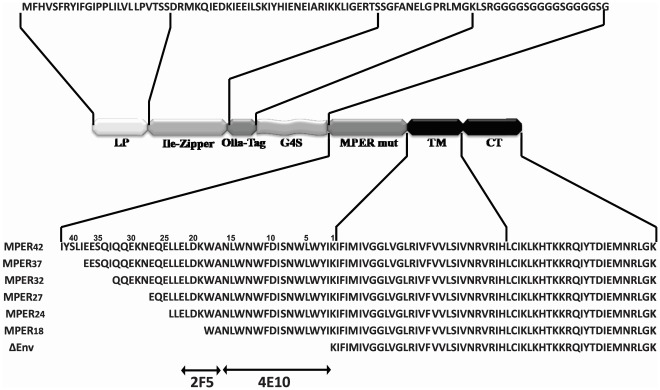
Map of domains and amino acid sequences of the MPER display mutants. The open reading frames of the MPER display mutants contain a heterologous leader peptide (LP), an isoleucine zipper to promote trimerization, a common tag (Ollas-tag) for easy detection, a flexible linker region (G4S), various regions of gp41 (MPERmut) including an HIV-1 transmembrane region (TM), and the cytoplasmic domain of the VSV-G protein (CT). The amino acid sequence of the different domains is given in the one-letter code. The numbering backward as depicted indicates the number of MPER-derived amino acid residues expressed by each mutant. The target regions for 2F5 and 4E10 monoclonal antibodies are indicated by double arrow-lines.

**Figure 2:**
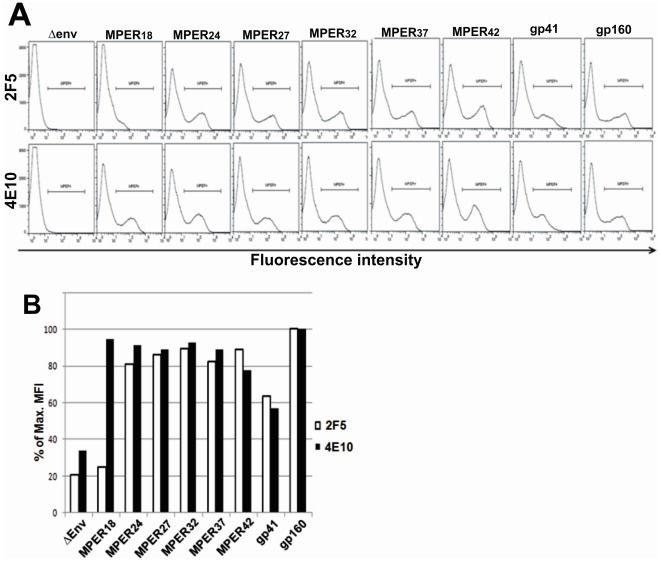
Surface expression of the MPER display mutants. (A) HEK293T cells transfected with expression plasmids encoding the different MPER display mutants were stained with 2F5 (top panel) and 4E10 (bottom panel) antibodies and analyzed by flow cytometry. Representative histograms of at least three independent transfection experiments are shown. (B) Mean fluorescence intensities (MFI) for the different MPER display mutants are expressed as percentage of the MFI obtained with gp160 Env.

### Confocal Microscopy of VLPs

GFP-labeled VLPs were prepared by co-transfection of HEK293T cells in 25 cm^2^ flask with 1 µg of the *gag-pol* expression plasmid HgpSyn and 1 µg of the *gag-GFP* expression plasmid pGag-eGFP (Cat. No. 11468, NIH AIDS reference and reagent program) with 2 µg of plasmids expressing either the MPER42 mutant, gp41, or R2 *env*. The supernatant (5 ml) was harvested 48 hours post transfection and cleared as described above. To analyze the MPER content of the VLPs, the supernatants were incubated for 30 minutes at room temperature under gentle shaking with 1 µg/ml of 4E10 antibody conjugated with Alexa Fluor 647 (Invitrogen, Oregon, USA). VLPs were recovered from the supernatant by ultracentrifugation through a 20% sucrose cushion and resuspended in 250 µl of 1×PBS. Slides for confocal analysis were prepared by mixing 5 µl of the resuspended VLPs with 5 µl of Prolong gold antifade reagent (Invitrogen, Oregon, USA) on clean 76×26 mm slides (Menzel GmbH, Braunschweig, Germany). The mixed drop was then carefully covered with an 18×18 mm cover-slide (Menzel GmbH, Braunschweig, Germany). The slide was kept in the dark for at least 24 hours prior to analysis on Leica DM IRE2 confocal laser scanning microscope (LSM) (Leica Microsystems GmbH, Wetzlar, Germany). Confocal fluorescent images were obtained using a 63× objective followed by a 4× digital magnification with the Leica confocal software. Green and red fluorescent images were collected after sequential excitation at 488 nm (eGFP) and 633 nm (Alexa Fluor 647). Images were then analyzed using the ImageJ software (NIH, http://rsbweb.nih.gov/ij/download.html) to generate overlays of the green and red fluorescent signals. This involved contrast enhancement of the images, but the same conditions were applied for all the images analyzed. The frequency of the co-localization of Alexa Fluor 647 positive spots with a GFP positive spot was determined by manual counting.

**Figure 3:**
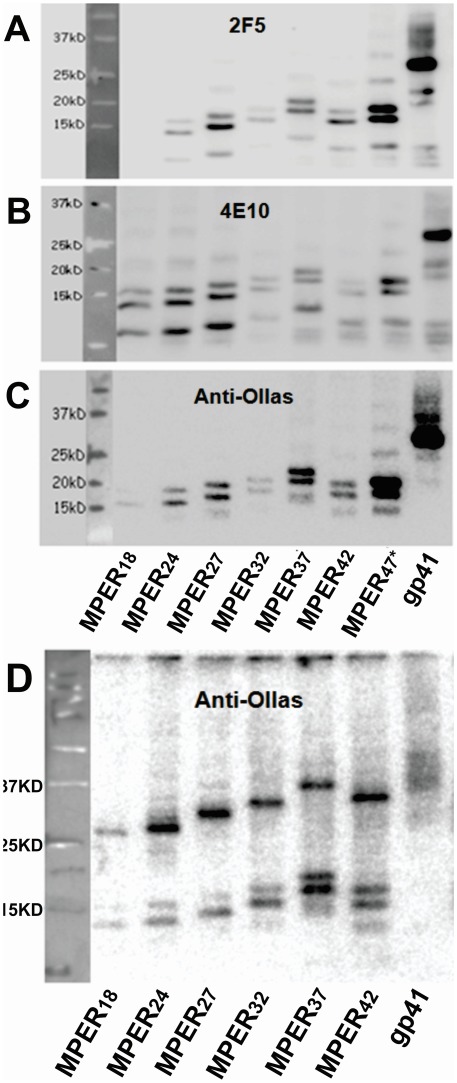
Incorporation of MPER display mutants into VLPs. VLPs containing MPER display mutants were concentrated from the supernatant of transfected cells and analyzed by Western blot analysis under denaturing (A to C) and non-reducing conditions (D) using the antibodies 2F5 (A), 4E10 (B), anti-Ollas (C, D). Results shown in Figures 3 A-D are representative of at least three WB analyses using different VLP preparations and different denaturing conditions. ^*^MPER47 contains 5 amino acids residue of non-MPER origin at its N-terminus.

**Figure 4:**
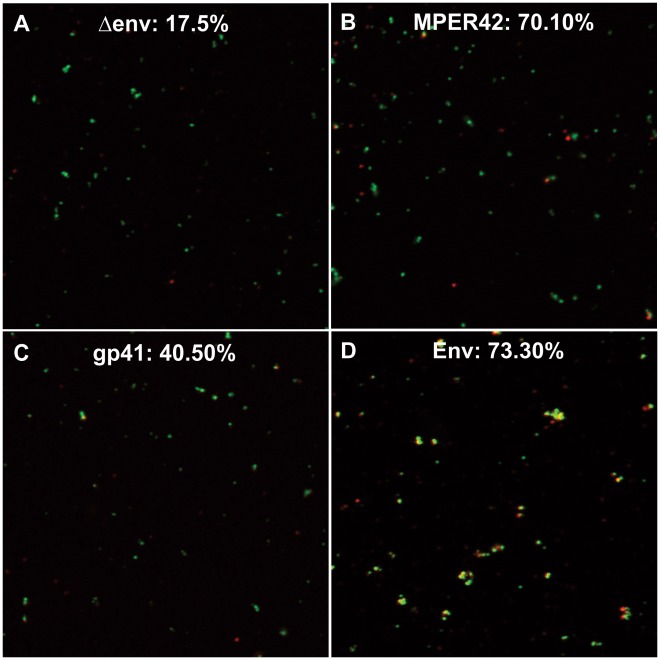
Co-localization of MPER42 with VLPs. VLPs were generated by co-transfection of the indicated *env* expression plasmids and a *gag-gfp* (green) expression plasmid and stained with 4E10 antibody. The percentage of red spots (n = 350 to 499 per sample) that co-localize with green spots is indicated.

### ELISA

#### Characterization of VLPs

The ELISA plates (LIA plates, Greiner Bio-one, Frickenhausen, Germany) were coated with the different VLP preparations or a recombinant gp41 standard (NIH AIDS Research and Reference Reagent Program, Germantown, USA) and detected using the 4E10 antibody and a HRP-labeled anti-human secondary antibody (Dako, Glostrup, Denmark). Relative light units (RLU) obtained with dilutions of gp41 standard were used to generate a calibration curve which enabled quantification of the MPER-Env content of VLP preparations.

#### Antibody detection in mouse serum

ELISA plates were coated with 200 ng/well of synthetic MPER peptides (NEQELLELDKWANLWNWFDISNWLWYIK, ProImmune, Oxford, UK), and blocked with 1X rotiblock (Carl ROTH, Karlsruhe, Germany). Serum was added at 1/100 dilution in 100 µl of 0.1× rotiblock, incubated 2 hours at RT. After 3 washing steps, bound antibodies were detected using polyclonal Rabbit anti-mouse-Ig HRP-labeled antibody (Dako, Glostrup, Denmark) and the ECL substrate. For the competitive ELISA, binding of a 1∶100 dilution of the sera in presence or absence of 8 µg/ml of 2F5 or 3D6 monoclonal antibodies was analyzed.

### DNA Electroporation

Female Balb/c mice were first anesthetized with 50 mg/kg of Ketamin and 10 mg/kg of Xylazin, and the hind legs shaved. The electroporator device (Ichor Medical Systems Inc, San Diego, USA.) was assembled as indicated by the manufacturer. The syringe containing the DNA solution (300 µg/ml formulated in 1×PBS) was inserted into position in the middle of the TriGrid™ electrode array, the array was inserted into the muscle of the shaved hind leg, and 50 µl of DNA solution was delivered followed by an electric pulse. The injection was repeated in the second hind leg, and each mouse received a total volume of 100 µl equivalent to 30 µg of plasmid DNA. Mice were vaccinated two or three times at 4 weeks interval.

### VLP Immunization

VLP aliquots were thawed from −80°C and diluted in 1×PBS to a concentration of 2 µg/ml of MPER Env-mutant or gp41. 50 µl of diluted VLP solution were injected subcutaneously into the footpad of each hind leg, thereby administering 200 ng of MPER protein per mouse. Mice were vaccinated three times at 4 weeks interval.

### Sampling

Three weeks after each immunization, mice were bled under general anesthesia by exposing them briefly to isofluran (Paragos, Frankfurt, Germany) vapors, and then 15 drops of blood were drawn from the retro-orbital sinus using a glass capillary tube. Blood samples were kept at RT for two hours, centrifuged at 4500 g and the serum was collected and stored at −20°C.

### Neutralization Assay in TZM-bl Cells

MN.3 clade B pseudotyped virus particles were produced by cotransfecting HEK293T/17 cells with 4 µg of an HIV-1 *rev/env* expression plasmid and 8 µg of an *env*-deficient HIV-1 backbone plasmid (pSG3deltaEnv) using Fugene6 transfection reagent (Roche, Mannheim, Germany). Plasmid details are given in the specimen catalog (number 4293) of the website of the HIV Specimen Cryorepository (GHRC): http://ghrc-portal.ibmt.fraunhofer.de/GHRC/welcome.do. Pseudovirus-containing supernatant was harvested 48 h following transfection and clarified by 0.45 µm filtration. Single-use aliquots (1.0 ml) were stored at −80°C. Virus stocks were diluted to an infectious dose yielding a RLU value of approximately 150,000 following infection of TZM-bl cells [27]. The virus particles were incubated with sera or control antibodies in a total volume of 100 µl for 1 hour at 37°C, and then TZM-bl cells diluted to 1×10^5^ cells per ml in DMEM medium supplemented with FCS (10%), gentamicin (50 µg/ml) and DEAE-Dextran (25 µg/ml) were added to each well. The plate was covered and incubated 48 hours at 37°C, 5% CO_2_ in a humidified atmosphere.

To measure the luciferase expression, supernatants were carefully removed and 50 µl of Glo lysis buffer (Promega GmbH, Mannheim, Germany) was added to each well and the plate was incubated 5 minutes at RT. The supernatant from lysed cells was pipetted up and down five times, and then transferred to a white microtiter plate (Greiner bio-one, Frickenhausen, Germany). 50 µl of Bright Glo reconstituted buffer were added to each well and the RLU was measured within 3 minutes.

**Figure 5:**
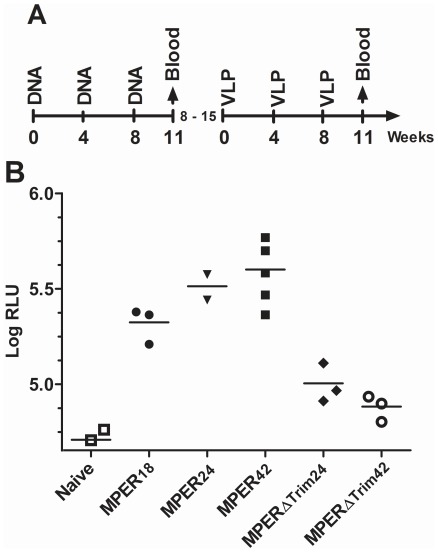
Antibody response after VLP boost in DNA immunized mice. (A) Mice were immunized three times with DNA encoding different MPER display mutants and boosted with VLPs containing the different MPER display mutants eight to fifteen weeks after the last DNA immunization. (B) MPER-specific antibody levels in the sera of immunized mice three weeks after the last VLP immunization are presented as log_10_ values of the relative light units (Log RLU) obtained in an MPER antibody ELISA. Mean and single values for each of the animals are shown.

**Figure 6:**
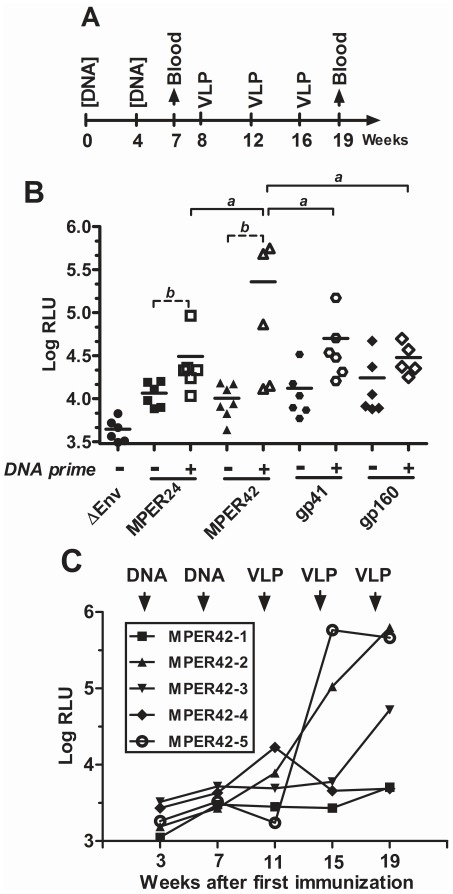
Influence of DNA priming on the MPER-specific antibody response after the VLP boost. (A) Mice (n = 5–6/group) were immunized with VLPs containing different MPER display mutant either with or without prior DNA immunization with plasmids encoding the same MPER display mutants. (B) MPER-specific antibody levels in the sera of immunized mice three weeks after the last VLP immunization are presented as Log values of the relative light units (Log RLU) obtained in an MPER antibody ELISA. Mean and single values for each of the animals are shown. Statistically significant differences between the groups treated with the different DNA and VLP vaccines were determined by one way analysis of variance followed by Bonferroni’s multiple comparison test and are indicated by horizontal bars with “a” indicating a p-value of <0.05. T-tests were performed to determine whether DNA priming enhances antibody levels for each of the different immunogens. Statistically significant differences are marked by dashed horizontal bars with “b” indicating a p-value of <0.05. Filled symbols are used for non-primed mice and opened symbols for DNA-primed mice. (C) Time course of MPER-specific antibodies in sera of individual mice (MPER42-1 to MPER42-5) immunized with MPER42 DNA and VLP vaccines.

## Results

### Design and Characterization of MPER Display Mutants

To focus the antibody response to MPER in its native conformation, large deletion mutants of gp41 expression plasmids were generated encoding only 18, 24, 27, 32, 37, or 42 of the most membrane proximal amino acids of the ectodomain of gp41 (Fig. 1). For efficient display of MPER on the cell membrane and on the surface of virus-like particles, the MPER sequences were flanked by a leader peptide at the N-terminus and a C-terminal transmembrane domain of HIV-gp41 fused to the intracytoplasmic domain of VSV-G. To further mimic the natural conformation and to facilitate detection of the different MPER mutants, a trimerization domain and a peptide tag (Ollas-Tag) were also included. The MPER sequences were separated from the N-terminal heterologous sequences by a flexible linker to facilitate folding of MPER into its native conformation.

To determine expression and accessibility of MPER, HEK293T cells were transfected with the different MPER display mutants and stained with 4E10 and 2F5 antibodies for flow cytometric analysis (Fig. 2A). The mean fluorescence intensities for cells transfected with the MPER display mutants were generally higher than those observed after transfection of the parental gp41 expression plasmid and similar in magnitude to the full-length Env (Fig. 2B). As expected from the deletion of the 2F5 core epitope, the MPER18 mutant was only detected by the 4E10 antibody, while expression of ΔEnv, which lacks the entire MPER could neither be detected by 4E10 nor by 2F5. The trimerization domain did not affect expression and accessibility, since transfection of MPER18, MPER24 and MPER42 mutants lacking the trimerization domain resulted in similar staining intensities as observed for the parental MPER display mutants (data not shown).

**Figure 7:**
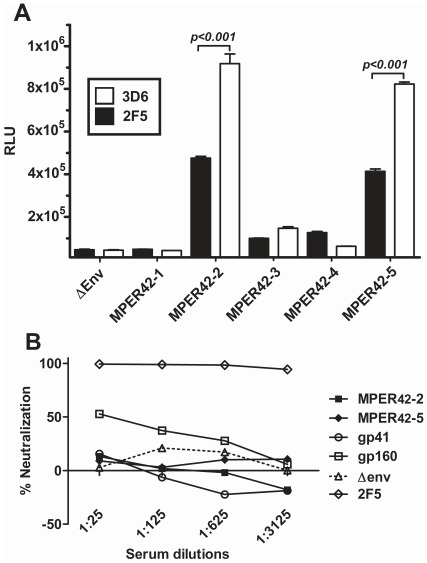
Characterization of the MPER-specific antibody response. (A) Binding of antibodies in sera of mice immunized with MPER42 by the DNA-VLP regimen to MPER peptide in the presence of saturating amounts of 2F5 and 3D6 monoclonal antibodies. Significant competition of 2F5 with serum antibodies from five individual mice (MPER42-1 to MPER42-5) for binding to MPER peptide is marked by the bars. (B) Neutralizing activity of sera of two individual mice with the strongest antibody response after immunization with MPER42 DNA and VLP vaccines. Sera from a ΔEnv control mouse and from mice responding strongest to immunization with gp41 and gp160 by the DNA-VLP regimen were also analyzed. The 2F5 antibody at a concentration of 50 µg/ml was diluted as indicated and used as a positive control for neutralization.

### Incorporation of MPER Display Mutants into VLPs

Since we aimed to analyze the immunogenicity of MPER display mutants by immunization with VLPs, the incorporation of the different MPER mutants into particles was analyzed. VLPs were partially purified from the supernatants of HEK293T cells cotransfected with HIV-1 *gag-pol* expression plasmid and the different MPER display mutants and MPER content was determined by Western blot analysis using 2F5, 4E10 and anti-Ollas antibodies (Fig. 3A-B-C).

The predicted molecular weight as calculated by Vector NTI program (Invitrogen) ranged between ∼18.5 kD for MPER18 and ∼21.3 kD for MPER42. None of the Western blot bands observed confirmed these predictions. For each of the MPER mutant three bands of higher electrophoretic mobility were observed (Fig. 3A-B-C). The apparent molecular weights of these three bands gradually increased with the numbers of MPER amino acids encoded by the MPER18 mutant up to the MPER37 mutant (Fig. 3A-B-C). For the MPER42 mutant, however, the electrophoretic mobility is substantially higher than the one observed for the MPER37 mutant. The results obtained are consistent for the three antibodies 2F5, 4E10, and anti-Ollas used for the detection of the MPER display mutants. Under non-reducing conditions, a larger band appears for all the mutants with approximately three times the apparent molecular weight of the smaller band obtained for each MPER mutant under stringent denaturing conditions (Fig. 3A-B-C). These results suggest substantial differences in electrophoretic mobility due to conformational differences associated with the high content of alpha-helical structures within MPER.

To obtain further evidence for incorporation of MPER mutants into VLPs rather than exosome like vesicles, the MPER42 mutant was cotransfected with a *gag-gfp* expression plasmid. The supernatant of the transfected cells was then incubated with fluorescently-labeled 4E10 antibody conjugated to Alexa Fluor 647. VLPs were pelleted through a 20% sucrose cushion and imaged by confocal microscopy. Co-localization of 70.1% of the Alexa Fluor 647 positive spots with GFP positive particles indicated incorporation of the MPER42 mutant into VLPs (Fig. 4B). For control VLPs lacking MPER a co-localization was observed in less than 17.5% of the Alexa Fluor 647 positive spots representing the background staining (Fig. 4A). The MPER42 mutant was incorporated with higher frequency than gp41 (40.5%) (Fig. 4C), and with comparable frequency as the wild type Env (73.3%) (Fig. 4D).

### Immunogenicity of MPER Display Mutants After DNA Prime-VLP Boost Immunization

Although the different MPER mutants did not differ substantially in accessibility to 4E10 and 2F5 (with the exception of MPER18), the Western blot analyses suggested different conformations. We therefore explored in a pilot immunization experiment in mice whether the MPER18, MPER24 and MPER42 mutants would induce different levels of MPER-specific antibodies. In addition, the MPER24 and MPER42 mutants lacking the trimerization domain (MPER24ΔTRIM, MPER42ΔTRIM) were included to explore a potential influence of the trimerization domain on the immunogenicity.

Mice were first immunized three times by intramuscular electroporation of DNA vaccines encoding the different MPER mutants (Fig. 5A). Although we had previously observed that two intramuscular electroporations of DNA vaccines encoding HA of influenza were sufficient to induce readily detectable levels of HA-specific antibodies [28], MPER-specific antibody responses remained undetectable even after the third DNA immunization. Therefore, the mice were further boosted three times by VLPs containing the same MPER display mutants used for the DNA immunizations (Fig. 5A). Three weeks after the last VLP immunization, MPER-specific antibody responses could be detected in all animals immunized with MPER display mutants containing the trimerization domain. In contrast, MPER specific antibody responses after immunization with MPER24ΔTRIM and MPER42ΔTRIM were only seen in a single mouse (Fig. 5B).

To confirm the induction of MPER-specific antibody responses and to determine the contribution of the DNA immunizations on the antibody responses seen after the VLP booster immunizations, mice were vaccinated with MPER24, MPER42, gp41 and full length Env gp160 containing VLPs with or without prior DNA priming immunizations (Fig. 6A).

As observed previously, DNA immunization alone did not induce detectable MPER-specific Ab response (data not shown and Fig. 6C). After the third VLP immunization, MPER-specific Ab responses were detectable in all animals that had been primed by DNA vaccination (Fig. 6B). VLP immunization alone also induced MPER-specific antibodies, but the levels were generally lower and some of the mice did not respond. As observed in the previous immunization experiment (Fig. 5B), the MPER42 display mutant tended to induce the highest MPER-specific antibody response (Fig. 6B). In two of the mice of the MPER42 group, the MPER-specific antibody response was more than 10-fold higher than the one observed following immunization with full-length gp160. For this group, MPER-specific antibodies were also analyzed longitudinally demonstrating a substantial increase in MPER-specific antibody levels after the second VLP immunization in two of the immunized mice (Fig. 6C).

### Characterization of the MPER-specific Antibody Response

To determine whether the MPER-specific antibodies induced by MPER_42_ DNA-VLP immunization could compete for binding with 4E10 and 2F5, sera were tested in the MPER peptide ELISA in the presence or absence of an excess of 4E10 and 2F5. The monoclonal antibody 3D6 binding to an epitope of gp41, which does not overlap with the MPER peptide used in the ELISA, was used as a negative control. Analyzing the sera with the highest MPER-specific antibody response, 2F5, but not 3D6 or 4E10 (data not shown) competed for binding to the MPER peptide (Fig. 7A) suggesting that antibodies were induced that either recognize or overlap the 2F5 epitope.

The same sera were also analyzed for neutralizing activity using the TZM-bl pseudotype assay [27] and compared to the neutralizing activity of sera from mice responding to DNA prime VLP boost immunization with gp41 or gp160 vaccines. No evidence of neutralization could be observed with the sera from MPER42 or gp41 immunized mice (Fig. 7B). Weak neutralization activity with a 50% neutralization titer of 1/25 was observed with the serum from the mouse immunized with gp160 vaccines.

## Discussion

Display of N-terminal deletion mutants of gp41 on the surface of cells increases 2F5 and 4E10 binding compared to gp41 (Fig. 2). This does not seem to be due to higher expression levels of MPER display mutants, since the VLPs contain at least as much of gp41 as of the MPER display mutants (Fig. 3). Thus, the accessibility of the MPER display mutants appears to be higher than the accessibility of gp41. To allow direct comparison of the properties of gp41 with those of the MPER display mutants, the gp41 was also expressed with an N-terminal leader sequence, the trimerization domain, the ollas-tag, and the flexible linker region. These modifications may explain why cells expressing wild type Env show higher binding of 2F5 and 4E10 than cells expressing the modified gp41. More importantly, the binding activity of 2F5 and 4E10 to cells expressing wild type Env was similar to cells expressing the MPER display mutants, confirming their efficient expression. Since 4E10 and 2F5 were selected for this study based on their efficient binding to MPER in the context of wild type Env, it is likely that the binding of these two monoclonal antibodies to MPER is not sterically blocked. Therefore, it is not expected that removing bulky domains can actually increase the binding of these two monoclonal antibodies. Both monoclonal antibodies contain unusual long complementary determining regions (CDR), which are important for their binding to MPER in the context of wild type Env [29]. Therefore, the removal of N-terminal domains of the gp41 ectodomain from immunogens might allow the induction of a much broader panel of MPER-specific antibodies including those lacking long CDRs.

The results of our immunization experiments clearly show that the MPER display mutants induce antibodies binding to the MPER peptide. The addition of a trimerization domain increased the MPER-specific antibody response, which might be either due to fixation of the MPER in its relevant native conformation or more efficient cross-linking of B-cell receptors leading to stronger activation of MPER-specific B cell clones. Immunization with the MPER42 display mutant induced in a subset of animals more than 10-fold higher anti-MPER antibody levels than immunization with wild type Env. The absence of other immunodominant epitopes in the MPER42 display mutant or better accessibility of the MPER42 display mutant for the B cell receptor on the surface of MPER-specific B cells may explain the observed difference in the MPER-specific antibody response. However, it should be noted that the strong MPER-specific antibody response was only observed in two of the five mice demonstrating substantial variability in the response to the same immunogen.

One important question is whether MPER display mutants induce antibodies binding to MPER in the context of wild type Env. Attempts to determine the binding activity of sera from mice immunized with MPER display mutants to wild type Env expressed on the surface of transfected cells were not successful (data not shown), since immunization with VLPs also induced antibodies against cellular proteins resulting in high background staining. Therefore, several possibilities remain, why the MPER peptide-specific antibodies induced by immunization with the MPER display mutants had no neutralizing activity. Firstly, the sensitivity of the MPER peptide ELISA could be higher than the sensitivity of the neutralization assay. Thus, the MPER-specific antibody levels induced might be too low to be detected in the neutralization assay. Secondly, the induced MPER-specific antibodies bind to MPER peptides but not to the native conformation of MPER. Given the design of the MPER-display mutants we consider this unlikely but cannot exclude this possibility. Thirdly, the MPER-specific antibodies induced by immunization with the MPER display mutants recognize MPER in its natural conformation, but their binding to MPER in the context of wild type Env is blocked by bulky domains of gp120 or gp41. Although we do not have direct evidence for the third possibility, this seems to be an inherent theoretical limitation of our approach and many other MPER peptide or mimotope-based HIV vaccination strategies. The neutralization mechanism of 2F5 and 4E10 antibodies have been described as a two-step “encounter-docking” process [5], [30] in which the antibodies weakly interact first with the lipid membrane, and then with its core-epitope extracted from the lipid membrane [5], [31]. The weak interaction involves the long hydrophobic CDR-H3 loop that characterizes the 2F5 and 4E10 broadly neutralizing antibodies [32]. The deletion of this CDR-H3 loop has been shown to abrogate the ability of 2F5 to neutralize HIV, but not to bind free MPER peptides [29], suggesting that the antibodies induced by vaccination should reproduce this particular CDR-H3 loop to possess the ability to neutralize.

After immunization with immunogens displaying an exposed MPER, high affinity antibodies to MPER can probably be raised without the need for long CDR-H3s. Thus, in the absence of a wild type Env immunogen there is no positive selection of B cells producing MPER-specific antibodies with long CDR-H3s. If it holds true, that long CDRs are required for binding of antibodies to MPER in the context of wild type Env, the induction of substantial levels of such antibodies by immunization with immunogens displaying an exposed MPER should be the exception rather than the rule.
